# Correction: AI-powered detection of cyberbullying in short-form video content: A hybrid deep learning framework

**DOI:** 10.1371/journal.pone.0350051

**Published:** 2026-05-26

**Authors:** Ahmad A. Mazhar, Islam Zada, Manal Aldhayan, Seetah Alsalamah, Mashael M. Asiri, Manel Ayadi, Abdullah Alshahrani

There are errors in the Acknowledgement section and the Funding section.

The correct Acknowledgement section is as follows: The research was supported by individual and combined research efforts of the following academic institutions: College of Communication, University of Sharjah, Sharjah, United Arab Emirates, Faculty of Computing & IT, International Islamic University Islamabad, Islamabad, Pakistan; College of Computer and Information Sciences, Shaqra University Shaqra 11961, Saudi arabia; College of Computer Science and Information, King Saud University, Riyadh, Saudi Arabia; Applied College at Mahayil, King Khalid University, Saudi Arabia. This research was supported by Princess Nourah bint Abdulrahman University Researchers Supporting Project number (PNURSP2026R761), Princess Nourah bint Abdulrahman University, Riyadh, Saudi Arabia, and Department of Computer Science and Artificial Intelligence, College of Computer Science and Engineering, University of Jeddah, Jeddah 21493, Saudi Arabia.

The authors would like to thank all the involved universities with their institutional support and cooperation during the research study and development of this research.

The correct Funding section is as follows: This research was supprted by Princess Nourah bint Abdulrahman University Researchers Supporting Project number (PNURSP2026R761), Princess Nourah bint Abdulrahman University, Riyadh,Saudi Arabia, and Department of Computer Science and Artificial Intelligence, College of Computer Science and Engineering, University of Jeddah, Jeddah 21493, Saudi Arabia.
