# Supplementary material for: AI-powered detection of cyberbullying in short-form video content: A hybrid deep learning framework
Source: PLoS One. 2026 Feb 11;21(2):e0338799. doi: 10.1371/journal.pone.0338799 (PMC12893605; doi:10.1371/journal.pone.0338799)
Supplement: Attachment — Submitted filename: renamed_b1bb6.docx [file pone.0338799.s003.docx]

**AUTHORS’ RESPONSE TO REVIEWERS**

**Journal Name:** PLOS ONE

**Manuscript ID:** PONE-D-25-36601

**Paper Title:** AI-POWERED DETECTION OF CYBERBULLYING IN SHORT-FORM VIDEO CONTENT: A HYBRID DEEP LEARNING FRAMEWORK

**Authors:** Ahmad A. Mazhar, Islam Zada, Manal Aldhayan, Seetah Alsalamah, Mashael M. Asiri, Manel Ayadi , Abdullah Alshahrani,

**General Response**

Thanks to the editor and four reviewers for their careful evaluation and insightful feedback. The manuscript has been **substantially revised** to address all scientific, structural, and stylistic concerns.
Major updates include:

- Condensed abstract with concise novelty and real-world relevance.
- Clearer articulation of motivation, gap, and contributions in the Introduction.
- Reorganized related work with critical comparisons and 2024-2025 citations.
- Expanded methodological detail (semantic-consistency layer, early stopping, new metrics – MCC and Cohen’s Kappa).
- Revised figures and tables with uniform decimals, improved captions, and percentage labels.
- Added discussion on limitations, deployment feasibility, and future work.
- All references standardized to **PLOS ONE citation format** with DOIs.

Page and section numbers refer to the revised manuscript (**V4-Final**).

All reviewer and editorial concerns have been carefully addressed. The revised manuscript now presents:

- A clearer motivation and contribution statement,
- Improved methodological transparency and statistical rigor, and
- Polished presentation consistent with **PLOS ONE** standards.

We sincerely thank the reviewers and editorial board for their valuable guidance, which has greatly improved the quality of our work.

A. **REVIEWER #1**

**Comment 1. Novelty of Semantic Consistency Validation Layer**

*How does this layer fundamentally differ from existing multimodal fusion approaches?*

**Response:**
We added a detailed explanation in **Section 3.3 (Semantic Consistency Validation, pp. 8–9)** describing that the layer applies **cross-modal self-attention with semantic-agreement loss Lₛ = 1 – cos(Fₜ,Fₐ,Fᵥ)** to enforce alignment across modalities. Unlike prior late-fusion models, this mechanism penalizes incongruent modality pairs and improves contextual coherence.

**Comment 2. Shortcomings of Prior Methods**

**Response:**
A new paragraph at the end of **Section 1 (Introduction, p. 4)** specifies limitations of existing methods – poor sarcasm detection, multimodal misalignment, and dataset imbalance – that our hybrid CNN-LSTM-Transformer framework directly addresses.

**Comment 3. Critical Comparative Analysis**

**Response:**
The **Literature Review (Section 2, pp. 5–7)** now includes a synthesized comparison highlighting technical gaps and tabular summary of feature-level fusion vs semantic-consistency methods (Refs [20]–[26]).

**Comment 4. Dataset Balance and Reliability**

**Response:**
Annotation reliability was verified using **Cohen’s κ = 0.87 (CAVD)** and **0.83 (SocialVidMix)**, added in **Section 3.4 (Data Collection and Preprocessing, p. 7)**.

**Comment 5. Statistical Significance**

**Response:**
We performed t-tests across ten random splits; all improvements were significant (p < 0.01). Results added beneath **Table 6 (pp. 17–18)**.

**Comment 6. Misclassified Samples and Future Work**

**Response:**
A new paragraph in **Section 5 (Discussion, p. 19)** links the error analysis (Table 4) to possible fusion-strategy refinements.

**Comment 7. Cross-Platform Experiments**

**Response:**
Clarified in **Section 4.6 (pp. 17–18)** that training used mixed-platform data (TikTok 60 %, Instagram 25 %, YouTube 15 %) and evaluated on unseen platform segments.

**Comment 8. Visualization Clarity**

**Response:**
All figures redrawn in high resolution (300 dpi) with clear axis labels and percentage annotations (Figures 4–9).

**Comment 9. Study Limitations**

**Response:**
Added new sub-section **“Limitations and Future Directions” (pp. 20–21)** discussing computational cost, language bias, and generalization.

**Reviewer #2**

**Comment 1. Condense Abstract**

**Response:**
Abstract shortened from 219 to 153 words; retained essential metrics and contributions (p. 1).

**Comment 2. Figure Captions Explain Trends**

**Response:**
Updated captions for Figures 2 & 3 to note “stable convergence and minimal overfitting.”

**Comment 3. Figure Numbering Consistency**

**Response:**
All figures captions standardized.

**Comment 4. Uniform Decimals in Tables**

**Response:**
Tables 3, 5 & 6 now display two-decimal precision.

**Comment 5. Dataset Names in Captions**

**Response:**
Captions of Tables 3–6 now explicitly list **CAVD** and **SocialVidMix** datasets.

**Comment 6. Keyword Repetition in Abstract**

**Response:**
Repetitive keywords merged with streamline phrasing.

**Comment 7. Recent Citations (2024–2025)**

**Response:**
Added new references [35-60] covering 2024–2025 developments in multimodal learning.

**Comment 8. Confusion Matrix Font Size**

**Response:**
Figure 4 enlarged and annotated with percentage values for readability.

**Comment 9. Reference Formatting**

**Response:**
All references now follow **PLOS ONE style**: italicized journal names, sentence-case titles, DOIs included where available.

**Reviewer #3**

**Comment 1. Practical Deployment Statement**

**Response:**
Added final paragraph in **Section 1 (p. 4)** describing real-time integration into moderation pipelines.

**Comment 2. Figure 4 & 7 Improvements**

**Response:**
Figure 4 updated with percentage labels; Figure 7 includes error bars showing variability across three runs.

**Comment 3. Uniform Decimal Precision**

**Response:**
All quantitative tables standardized (see Tables 3–6).

**Comment 4. Early Stopping Clarification**

**Response:**
Specified that early stopping based on validation-loss plateau was applied across all modalities (Section 3.5 p. 8).

**Comment 5. Additional Metrics (MCC/Kappa)**

**Response:**
Reported in **Table 6** and discussed in **Results (p. 17)** to strengthen evaluation on imbalanced data.

**Comment 6. Low-Resource Language Extension**

**Response:**
Added note in **Conclusion (p. 20)** regarding future adaptation to non-English datasets.

**Comment 7. Reference Style and Completeness**

**Response:**
Reference list fully standardized with consistent numbering [17, 23, 31 re-indexed].

**Comment 8. Limitations Acknowledged**

**Response:**
New paragraphs in **Discussion (p. 20-22)** recognize scalability and computational-resource limits.

**Comment 9. Validation Justification**

**Response:**
Section 4.5 expanded to explain 5-fold cross validation and statistical averaging across runs.

**Comment 10. Future Research Directions**

**Response:**
Outlined in **Conclusion (pp. 20–21)** including real-time streaming integration and explainable-AI extension.

**Reviewer #4**

**Comment 1. Technical Detail in Abstract**

**Response:**
Abstract now explicitly mentions datasets (CAVD & SocialVidMix) and main performance (Accuracy = 91.6 %, F1 = 91.3 %).

**Comment 2. Motivation and Challenges in Introduction**

**Response:**
Rewrote **first three paragraphs of Section 1 (pp. 2–3)** to highlight deficiencies of prior approaches and specific challenges addressed.

**Comment 3. Explicit Contributions**

**Response:**
Added bullet-point list of four contributions at the end of **Introduction (p. 4)**.

**Comment 4. Organizing Related Work**

**Response:**
Sub-headings introduced: “Text-Based Approaches,” “Visual/Audio Approaches,” and “Hybrid Multimodal Approaches” (Section 2).

**Comment 5. Comparative Study**

**Response:**
Inserted comparative discussion paragraph with Table 2a summarizing prior techniques vs proposed method.

**Comment 6. Grammar and Language Polish**

**Response:**
Entire manuscript professionally proof-read and grammatically standardized.

**Comment 7. Algorithm Selection Rationale**

**Response:**
Section 3.2 (Feature Extraction) now justifies CNN for spatial cues, LSTM for temporal audio, Transformer for textual semantics, with supporting citations.

**Comment 8. Confusion Matrix Explanation**

**Response:**
Clarified that Figure 4 represents results from the **proposed hybrid model**; accuracy now matches Table 3 values.

**Comment 9. Study Limitations**

**Response:**
Comprehensive paragraph in **Discussion (p. 20-23)** elaborates dataset scope, computational overhead, and ethical considerations.
